# Inferring Muscle-Tendon Unit Power from Ankle Joint Power during the Push-Off Phase of Human Walking: Insights from a Multiarticular EMG-Driven Model

**DOI:** 10.1371/journal.pone.0163169

**Published:** 2016-10-20

**Authors:** Eric C. Honert, Karl E. Zelik

**Affiliations:** 1 Department of Mechanical Engineering, Vanderbilt University, Nashville, Tennessee, United States of America; 2 Department of Biomedical Engineering, Vanderbilt University, Nashville, Tennessee, United States of America; 3 Department of Physical Medicine and Rehabilitation, Vanderbilt University, Nashville, Tennessee, United States of America; Fondazione Santa Lucia Istituto di Ricovero e Cura a Carattere Scientifico, ITALY

## Abstract

**Introduction:**

Inverse dynamics joint kinetics are often used to infer contributions from underlying groups of muscle-tendon units (MTUs). However, such interpretations are confounded by multiarticular (multi-joint) musculature, which can cause inverse dynamics to over- or under-estimate net MTU power. Misestimation of MTU power could lead to incorrect scientific conclusions, or to empirical estimates that misguide musculoskeletal simulations, assistive device designs, or clinical interventions. The objective of this study was to investigate the degree to which ankle joint power overestimates net plantarflexor MTU power during the Push-off phase of walking, due to the behavior of the flexor digitorum and hallucis longus (FDHL)–multiarticular MTUs crossing the ankle and metatarsophalangeal (toe) joints.

**Methods:**

We performed a gait analysis study on six healthy participants, recording ground reaction forces, kinematics, and electromyography (EMG). Empirical data were input into an EMG-driven musculoskeletal model to estimate ankle power. This model enabled us to parse contributions from mono- and multi-articular MTUs, and required only one scaling and one time delay factor for each subject and speed, which were solved for based on empirical data. Net plantarflexing MTU power was computed by the model and quantitatively compared to inverse dynamics ankle power.

**Results:**

The EMG-driven model was able to reproduce inverse dynamics ankle power across a range of gait speeds (R^2^ ≥ 0.97), while also providing MTU-specific power estimates. We found that FDHL dynamics caused ankle power to slightly overestimate net plantarflexor MTU power, but only by ~2–7%.

**Conclusions:**

During Push-off, FDHL MTU dynamics do not substantially confound the inference of net plantarflexor MTU power from inverse dynamics ankle power. However, other methodological limitations may cause inverse dynamics to overestimate net MTU power; for instance, due to rigid-body foot assumptions. Moving forward, the EMG-driven modeling approach presented could be applied to understand other tasks or larger multiarticular MTUs.

## Introduction

**Fig 1:**
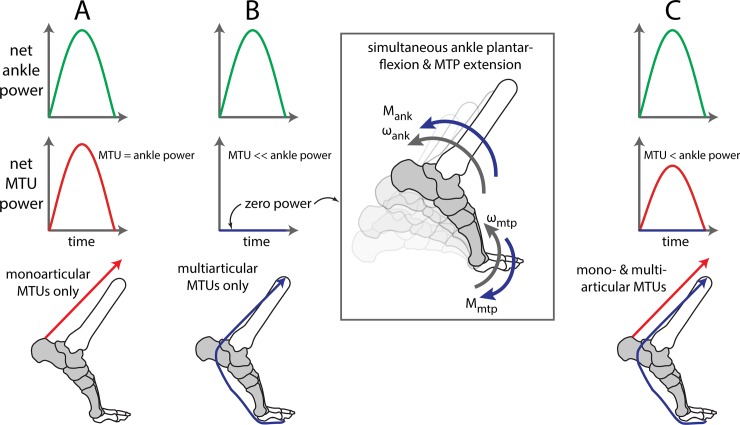
Conceptual summary of ankle joint vs. muscle-tendon-unit (MTU) power. Net ankle joint power (green, top row) can be computed from inverse dynamics by multiplying ankle joint moment, *M*_*ank*_, by ankle angular velocity, *ω*_*ank*_ (sagittal plane depicted). Due to assumptions in inverse dynamics, this ankle power may or may not correspond with net MTU power (second row), depending on the underlying MTU contributions (cartoon depicted in bottom row). (A) Ankle power is expected to reflect MTU power when MTUs are monoarticular (red, acting solely about the ankle). (B) Ankle power may not reflect power contributions from multiarticular MTUs (blue). In the extreme example depicted, the multiarticular MTU provides torque about both the ankle and MTP (toe) joints, but due to the simultaneous plantarflexion of the ankle and extension of the toes, the MTU does not change length. Thus the MTU behaves like a rigid cable and performs zero net power. However, inverse dynamics (joint-by-joint) analysis would indicate positive power about the ankle (*M*_*ank*_ ∙ *ω*_*ank*_), and equal offsetting negative power about the toe joints (*M*_*MTP*_ ∙ *ω*_*mtp*_, inset). In this case, net ankle power would greatly overestimate net MTU power. (C) In actuality, both mono- and multi-articular MTUs contribute to human movement. However, it remains unclear if and by how much ankle power overestimates net MTU power. If multiarticular MTUs act isometrically (i.e., perform zero net power, as depicted here) or close to isometrically, then it is expected that ankle power magnitude will be larger than net MTU power.

Inverse dynamics estimates of joint kinetics are often used to infer contributions from underlying groups of muscle-tendon units (MTUs), allowing the use of external force and motion recordings to gain insight into musculoskeletal biomechanics and neuromuscular coordination [1]. For instance, net joint moments (also called torques) or trends in net joint moments are commonly interpreted as a surrogate for net torques created by groups of MTUs about a joint [2]. This interpretation hinges on a number of assumptions, such as negligible torque contributions from non-MTU sources (e.g., ligaments). Similarly, net joint power (computed by multiplying net joint moment by joint angular velocity) can be interpreted as a reflection of net power generated by groups of MTUs crossing a joint [2–4]. However, this interpretation is based on the assumption that joint power originates entirely from monoarticular MTUs, which cross only a single joint (Fig 1A). When multiarticular (multi-joint) MTUs generate force or power during movement, then inverse dynamics joint power may over- or under-estimate the actual net MTU power (Fig 1), due to inadvertently subdividing power from a single multiarticular MTU into positive power at one joint and offsetting negative power at another. This methodological issue confounds our ability to infer MTU dynamics from traditional motion analysis. In practice, over- or under-estimating MTU power could lead to incorrect scientific conclusions, or to empirical estimates that misguide musculoskeletal simulations, assistive device designs, or clinical interventions. It is therefore critical to evaluate and understand the degree to which inverse dynamics joint power estimates reflect underlying MTU power.

We investigated the hypothesis that inverse dynamics ankle power overestimates net plantarflexor MTU power during the end of stance phase in human walking, termed Push-off, due to neglected multiarticular ankle-foot MTU dynamics. This objective was intentionally specific to a single joint, a subset of MTUs (the ankle plantarflexors), and a single phase of gait (Push-off). This is because interpreting net MTU power from joint power is highly task- and joint-specific. For instance, if for one locomotor task the multiarticular MTUs that cross the ankle and metatarsophalangeal (MTP, i.e. toe) joints are largely unloaded (i.e., contribute little force), then inverse dynamics joint power estimates may provide a reasonable approximation of the net MTU power. However, for a different locomotor task the multiarticular MTUs may generate substantial force and power, and confound our ability to infer net MTU power directly from joint-level estimates (Fig 1B & 1C). At the same time, other multiarticular MTUs that cross the knee and hip (e.g., hamstrings) may be behaving completely differently. These joints/muscles would require their own separate task-specific assessment of whether joint power is reflective of net MTU power (for a subset of muscles crossing a joint). In light of this task- and joint-specificity we focused this study solely on MTUs that directly contribute to ankle joint plantarflexion.

Ankle plantarflexors were studied because these MTUs are major power producers during walking [5,6]. The majority of this power is produced in a burst-like fashion during the Push-off phase of walking, which occurs during roughly 45–65% of the stride cycle, immediately before the foot lifts off the ground [7]. Push-off helps accelerate the leg into swing [6,8,9] and redirect/accelerate the body’s center-of-mass, which can potentially reduce collisional energy losses after contralateral foot contact and thereby facilitate economical gait [10,11].

Inverse dynamics calculations are commonly used to estimate power about a single joint, but do not account for the behavior of multiarticular musculature, such as the flexor digitorum and hallucis longus (FDHL) MTUs, which cross the ankle and MTP joints. Based on published ankle and toe kinematics [12] and the multiarticular FDHL functions [13,14], we expect that inverse dynamics ankle power overestimates net positive MTU power generation during Push-off. Conceptually, this scenario is depicted in Fig 1C. As a direct consequence, the amount of negative power absorbed by the foot may also be overestimated, which could explain why the foot appears to undermine ankle Push-off by absorbing energy [15]. Misestimating ankle or foot kinetics could affect our understanding of where power is generated/absorbed in the body during human gait, which has implications on musculoskeletal simulations that rely (directly or indirectly) on empirical kinetics estimates, and on assistive devices (e.g., foot prostheses) that are often designed to mimic biological function. For example, overestimating biological ankle power could result in powered prostheses that are designed with more powerful actuators and heavier batteries than necessary. However, the magnitude of the power overestimate is currently unknown, as it requires us to account for multiarticular MTU contributions.

Accounting for multiarticular power contributions requires the ability to parse out individual MTU kinetics. Biomechanical estimates such as inverse dynamics are derived from empirical recordings and an underlying link-segment model; however, these can only approximate net joint kinetics, and cannot resolve individual MTU contributions [1,16]. More direct measurements would require a comprehensive set of implantable force and strain sensors, which are impractical for in vivo human experiments. In lieu of direct measures, one common alternative is to develop a musculoskeletal model to approximate individual MTU contributions [14,16–19]. In this study we implemented an electromyography (EMG) driven musculoskeletal model using an EMG-to-force mapping algorithm, which allowed us to approximate individual MTU contributions to gait. We then used this approach to investigate ankle-foot interplay during human walking, and specifically to evaluate the hypothesis that ankle joint Push-off power overestimates net plantarflexor MTU power due to neglected multiarticular dynamics.

## Methods

We employed an EMG-driven musculoskeletal model to determine if inverse dynamics joint power overestimates the net MTU power during the Push-off phase of walking, due to inadvertently subdividing multiarticular MTU power into positive power at one joint (ankle) and offsetting negative power at another (MTP). We conducted a human gait analysis experiment to collect kinematics, kinetics, and surface EMG data. First we computed joint power using a standard inverse dynamics approach. Then, anthropometric and empirical data were combined via a musculoskeletal model to estimate the power contributions from mono- and multi-articular MTUs that cross the ankle. Net MTU power (and work) was then compared to inverse dynamics estimated joint power (and work).

### Experimental Data Collection

Six healthy subjects (3 males and 3 females, mean±standard deviation, 24±5 years, 88±14 kg, 1.8±0.1 m height) completed a gait analysis study. All subjects gave informed consent to the protocol, which was approved by the Institutional Review Board at Vanderbilt University. The study was performed over two days. The first day was a subject screening session that involved training and verification of EMG signals. Data were collected the second day, to avoid confounds due to muscle fatigue.

The purpose of the screening session (day 1) was to determine if we could independently record surface EMG from the ankle plantarflexor muscles, without excessive signal cross-talk [20]. Delsys Trigno surface EMG sensors (Delsys, Natick, MA, USA) were placed unilaterally on the participant’s major ankle plantarflexor muscles, specifically the triceps surae (soleus, medial gastrocnemius, and lateral gastrocnemius) and peroneus longus, according to the recommendations of the Surface Electromyography for the Non-Invasive Assessment of Muscles project (seniam.org), a European project on surface EMG [21]. An additional surface EMG sensor, a Delsys Trigno Mini, was placed above the ipsilateral flexor digitorum and hallucis longus (FDHL) muscles. The FDHL muscles are multiarticular in nature and contribute to ankle plantarflexion, longitudinal arch support, and MTP flexion. Due to the proximity of the flexor digitorum longus and flexor hallucis longus muscles at the EMG sensing site, the EMG signals were measured together, similar to prior literature [20,22,23]. These plantarflexing muscles were chosen based on their accessibility via surface EMG. Based on our prior experience recording ankle-foot muscles, the primary concern was with soleus muscle cross-talk affecting the FDHL recording [20]. Subjects were given visual biofeedback of the muscle EMG signals, and asked to activate soleus vs. FDHL separately. If the subject could independently activate their soleus muscle without exhibiting FDHL EMG activation, then they were invited to participate in the day 2 data collection. Six of the seven subjects initially recruited were able to demonstrate separate soleus vs. FDHL signals, and thus these six individuals completed the second day experimental protocol.

The gait analysis session (day 2) involved participants walking on an instrumented treadmill in a motion capture space while collecting EMG signals. We placed surface EMG sensors in the same locations detailed for day 1 and again verified the sensor placement through the day 1 protocol. In addition, 19 retro-reflective motion capture makers were affixed to the subject’s ipsilateral shank and foot, similar to marker sets used in prior foot studies [24,25], so that ankle and MTP joints, and longitudinal arch motion could be estimated. Prior to walking trials, subjects performed a set of quasi-static maximum voluntary contraction trials against manual resistance which targeted each of the muscles recorded via EMG. Next, subjects performed walking trials at three speeds (0.75, 1.00 and 1.25 m/s) on a force instrumented split-belt treadmill (Bertec, Columbus, OH, USA), while we recorded ground reaction forces under each foot at 2000 Hz, EMG at 2000 Hz, and kinematics at 100 Hz (Vicon T40, Oxford, UK).

### Experimental Data Processing

Post-processing was performed using Visual3D software (C-Motion, Germantown, MD, USA) and custom MATLAB (MathWorks, Natick, MA, USA) code. Marker and force data were low-pass filtered at 6 Hz and 15 Hz, respectively. EMG signals were demeaned, high-pass filtered at 150 Hz, rectified, low-pass filtered at 10 Hz [20,26], and then normalized based on the maximum muscle activation magnitude that occurred throughout all of the recorded trials (i.e., both walking and maximum voluntary contraction trials). All filters used were 3^rd^ order, zero-lag Butterworth filters. Foot and ankle kinematics were estimated from the motion capture data. Inverse dynamics was used to compute sagittal ankle power. We divided and averaged across strides (from footstrike to ipsilateral footstrike), to obtain subject-specific kinematics, kinetics, and EMG waveforms. We used these stride-averaged waveforms as inputs to a musculoskeletal model we developed (implemented in MATLAB), to estimate sagittal plane power contributions from individual ankle-foot MTUs. For reporting and comparison purposes, we computed the peak Push-off power and Push-off work, the latter by integrating underneath the positive region of the ankle power curve in late stance.

### Musculoskeletal Model

We utilized a simple EMG-to-force mapping algorithm to estimate MTU-specific contributions to ankle plantarflexion. The model used was an extension of methods published by Farris and Sawicki (2012). For each muscle, *m*, we used the EMG envelope (*EMG*_*m*_), muscle size (*PCSA*_*m*_, [27]), and fiber pennation angle (*θ*_*m*_, [27]) to estimate an unscaled, time-varying force profile. *EMG*_*m*_, the normalized EMG signal for each muscle, was defined as a function of time (*t*) and electromechanical delay (EMD, *τ*). To obtain a MTU force estimate, *F*′_*m*_, in units of Newtons per kilogram we needed an additional subject- and speed-specific constant, *C* (Eq 1). This constant accounts for electrophysiological differences between subjects as well as differences in contraction dynamics across speeds. Note that “prime” variables (e.g., $F_{m}^{\prime }$) are all obtained from the EMG-driven model.

$${F\prime }_{m}(t)={PCSA}_{m}∙\cos\left(\theta_{m}\right)∙{EMG}_{m}(t-\tau )∙C$$ (1)

Next, we computed inverse dynamics ankle power, *P*_*ank*_, from experimental motion and force data. We then combined *F*′_*m*_ with published moment arms for each muscle [13] to estimate the relative moment contributions from individual MTUs about the ankle joint, $M_{m,ank}^{\prime }$. We used these EMG-driven model estimates to approximate ankle joint power ($P_{ank}^{\prime }$), by multiplying the summed MTU moment waveform ($\sum \;M_{m,ank}^{\prime }$) by the sagittal plane ankle angular velocity, *ω*_*ank*_, which was computed from motion capture data (Eq 2). The EMD, *τ*, was estimated through a cross-correlation analysis that sequentially shifted the MTU moment waveform in time, multiplied by *ω*_*ank*_, and then found the correlation between the resultant waveform and inverse dynamics ankle power, *P*_*ank*_. The time lag associated with the maximum correlation was defined as the EMD for all muscles at a given speed. The power computed using this EMD was then scaled to the peak inverse dynamics ankle power, *P*_*ank*_, through the use of a speed- and subject- specific scaling factor *C*, to yield $P_{ank}^{\prime }$, which was reported in the units of W/kg. $P_{ank}^{\prime }$ was then decomposed into individual MTU power contributions. We performed additional computations to account for multiarticular MTU behaviors (as detailed below in the section “Estimating MTU Power”) and to thereby test our hypothesis that inverse dynamics ankle joint Push-off power overestimates the net power provided by MTUs crossing the ankle. Since the model we used was reasonably similar to previously published approaches we elected to only briefly summarize here; however, for completeness and reproducibility full model details are presented in S1 Appendix.

$${P^{\prime }}_{ank}(t)=\sum_{m}P_{m,ank}^{\prime }(t)=\sum_{m}M_{m,ank}^{\prime }(t)∙\omega_{ank}(t)$$ (2)

### Model Evaluation

We performed a model evaluation prior to investigating our hypothesis. We calculated the correlation (coefficient of determination, R^2^) between the scaled model-estimated ankle power, $P_{ank}^{\prime }$, and inverse dynamics estimated ankle power, *P*_*ank*_, at each walking speed. We would lack confidence in our musculoskeletal model if it was unable to reasonably reproduce ankle kinetics. Additionally, we examined how model computed coefficients (*C* and *τ*) varied with speed. Based on prior literature, we expected the EMD to decrease with increasing speed because as speed increases, MTU force increases [28], and as MTU force increases, EMD has been observed to decrease [29]. We also anticipated that various factors could cause the scaling factor *C* to be non-constant across gait speeds. For example, as speed increases, plantarflexing muscle contractions during Push-off become more concentric (i.e., less isometric) [30], which affects the mapping of force-to-EMG [31].

### Estimating MTU Power

We estimated net plantarflexing MTU power by examining the contributions of the multiarticular FDHL in two different ways: first for a simplified case by assuming the multiarticular FDHL MTUs act isometrically (i.e., MTUs performed zero net power), and second by estimating FDHL power from ankle and foot kinematics (similar to [24]). The first case was useful to assess the worst case scenario, i.e., when the net MTU power from the ankle plantarflexors would be smallest in magnitude and, therefore, inverse dynamics would be expected to maximally overestimate net MTU power. This would occur if the multiarticular FDHL MTUs performed no mechanical work (i.e., MTUs acted like a rigid cable). In our model, we computed this minimum MTU power (*P*′_*MTU*,*min*_) by subtracting the FDHL power due to ankle rotation (*M*′_*fdhl*,*ank*_ ∙ *ω*_*ank*_) from the model-estimated net ankle power $P_{ank}^{\prime }$ (Eq 3).

$${P^{\prime }}_{MTU,min}(t)=P_{ank}^{\prime }(t)-{P^{\prime }}_{fdhl,ank}(t)$$ (3)

**Fig 2:**
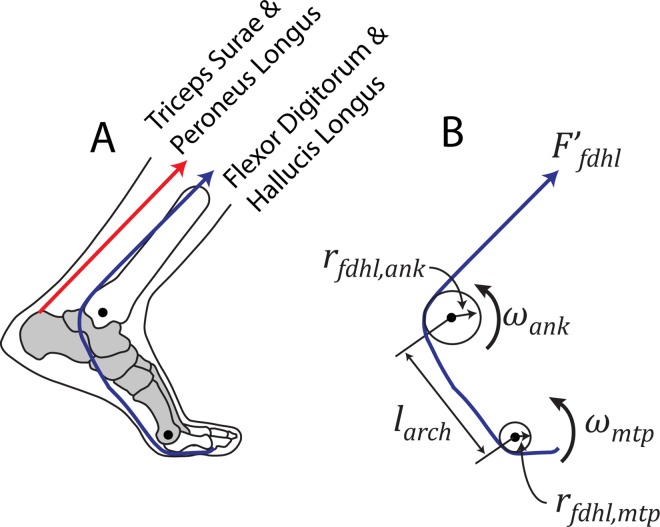
Simplified representation of ankle-foot musculoskeletal model. This simplified model was used to investigate the ankle plantarflexor muscles during the Push-off phase of walking. (A) The main ankle plantarflexor MTUs were included in the model: triceps surae (soleus and gastrocnemius), the peroneus longus, and the flexor digitorum and hallucis longus (FDHL). See S1 Appendix for more details on muscles that were included/excluded. (B) Kinematic, anthropomorphic, and EMG data were used to estimate power contributions from each MTU. An example is depicted for the multiarticular FDHL MTUs. Anthropomorphic MTU moment arms about the ankle (*r*_*fdhl*,*ank*_) and MTP joints (*r*_*fdhl*,*mtp*_) were combined with kinematic estimates–angular velocities of the ankle (*ω*_*ank*_) and MTP joints (*ω*_*mtp*_), and longitudinal arch length (*l*_*arch*_)–to estimate time-varying MTU length changes. MTU force was estimated using an EMG-to-force mapping algorithm (see S1 Appendix for full details). Force was then multiplied by the rate of MTU length change to compute MTU power.

Next, we estimated net MTU power from the ankle plantarflexors without the simplifying (isometric MTU) assumption. Instead we took into account the entire MTU excursion by combining kinematic estimates of angular velocities (*ω*_*ank*_ and *ω*_*mtp*_), moment arms (*r*_*fdhl*,*ank*_ and *r*_*fdhl*,*mtp*_ reported in [13,32,33]), and longitudinal arch length, *l*_*arch*_ [24,25], see Fig 2) to estimate power from the multiarticular FDHL MTUs. We first estimated FDHL power contributions about the ankle only (Eq 4), then FDHL power contributions due to motion within the foot (Eq 5), and finally summed these to obtain a complete estimate of the net FDHL MTU power (Eq 6). As an intermediate computation, the rate of FDHL length change due to ankle rotation was attributed to ankle power (*P*′_*fdhl*,*ank*_, Eq 4), and the rate of change due to longitudinal arch lengthening/shortening and to MTP joint rotation were attributed to foot power (*P*′_*fdhl*,*foot*_, Eq 5). We then defined the summation of these quantities as the net FDHL MTU power (*P*′_*fdhl*_, Eq 6). This estimate reflects power generated or absorbed by the multiarticular FDHL MTUs themselves rather than estimated contributions about a single joint. This formulation avoids the problem in which a multiarticular MTU’s power may be inadvertently subdivided into positive power at one joint and offsetting negative power at another.

$${P^{\prime }}_{fdhl,ank}(t)=\omega_{ank}(t)∙r_{fdhl,ank}∙{F^{\prime }}_{fdhl}(t)$$ (4)

$${P^{\prime }}_{fdhl,foot}(t)=\omega_{mtp}(t)∙r_{fdhl,mtp}∙{F^{\prime }}_{fdhl}(t)+\frac{d}{dt}(l_{arch}(t))∙{F^{\prime }}_{fdhl}(t)$$ (5)

$${P^{\prime }}_{fdhl}(t)={P^{\prime }}_{fdhl,ank}(t)+{P^{\prime }}_{fdhl,foot}(t)$$ (6)

*P*′_*fdhl*_ could then be added to other plantarflexing MTU powers obtained through the musculoskeletal model in order to estimate the net MTU plantarflexing power (*P*′_*MTU*_, Eq 7). This assumes that power from each other plantarflexor MTU can be approximated by calculating *M*′_*m*_ ∙ *ω*_*ank*_; in other words, we assumed that *P*′_*m*,*ank*_ = *P*′_*m*_ for each of the other MTUs. This is expected for monoarticular MTUs (e.g., soleus) acting only about the ankle joint. This assumption was also applied to the gastrocnemius (multiarticular ankle-knee) MTUs. During the Push-off phase of walking the ankle is plantarflexing while the knee is flexing, which both contribute to gastrocnemius MTU shortening. Since both motions are indicative of positive gastrocnemius MTU power, we would not predict positive power at one joint and offsetting negative power at the other.

$${P^{\prime }}_{MTU}(t)=-{P^{\prime }}_{fdhl,ank}(t)+\sum_{m}{P^{\prime }}_{m,ank}(t)={P^{\prime }}_{fdhl,foot}(t)+\sum_{m}{P^{\prime }}_{m,ank}(t)$$ (7)

### Musculoskeletal Model Sensitivity to EMG

In order to assess the EMG-driven model sensitivity to maximum muscle activations, we performed an additional Monte Carlo simulation using data from one subject. This simulation randomly varied the maximum activation level for each muscle between 50% and 150% of its nominal value, and we computed net MTU work for 1000 iterations.

## Results

**Fig 3:**
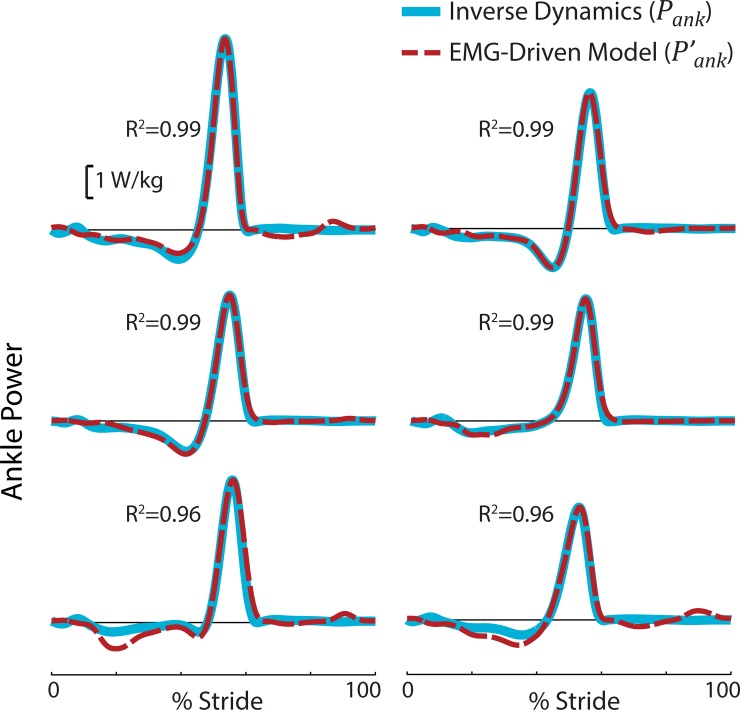
EMG-driven musculoskeletal model was able to reproduce inverse dynamics sagittal plane ankle power. Results are depicted for each individual subject at 1.25 m/s. The EMG-driven ankle joint power, $P_{ank}^{\prime }$, (red dashed line) correlated strongly with inverse dynamics ankle power, *P*_*ank*_, (blue solid line).

We found that the EMG-driven model-estimated ankle power, $P_{ank}^{\prime }$, correlated strongly with inverse dynamics ankle power (*P*_*ank*_, R^2^ = 0.98 ± 0.02 at nominal speed of 1.25 m/s, Fig 3). This finding was consistent across walking speeds tested (R^2^ = 0.97 ± 0.02 at 0.75 m/s, R^2^ = 0.97 ± 0.04 at 1 m/s). The values reported are inter-subject means and standard deviations.

On average, the subject-specific scaling factor, *C*, and the EMD, *τ*, both decreased from 0.75 to 1.25 m/s (Table 1). However, there was intersubject variability, and each of these decreasing trends was observed in only 4 of the 6 subjects. EMD ranged from about 30–130 ms across subjects and speeds, and the scaling factor ranged from about 1.8 to 2.9 N/kg·cm^2^.

**Table 1:** Electromechanical delay (EMD, *τ*) and scaling factor, *C*, for each speed, subject, and the overall study average.

Subject	Speed					
	0.75 m/s		1.00 m/s		1.25 m/s	
	EMD, *τ* (sec)	Scaling Factor, *C* (N/kg·cm^2^)	EMD, *τ* (sec)	Scaling Factor, *C* (N/ kg·cm^2^)	EMD, *τ* (sec)	Scaling Factor, *C* (N/ kg·cm^2^)
1	0.060	2.80	0.064	2.40	0.062	1.80
2	0.082	2.60	0.061	2.21	0.038	1.83
3	0.106	2.03	0.087	2.29	0.077	2.12
4	0.100	2.29	0.079	2.21	0.060	1.97
5	0.050	2.19	0.052	2.93	0.061	2.08
6	0.129	1.89	0.078	2.18	0.034	2.07
Average	0.088	2.30	0.070	2.37	0.055	1.98

**Fig 4:**
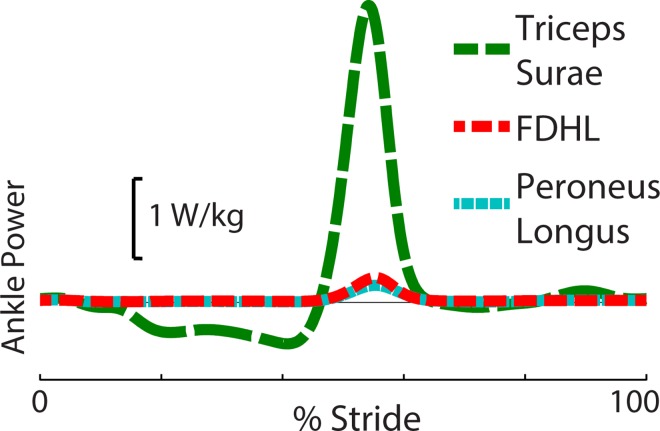
Average MTU contributions to $P_{ank}^{\prime }$ at 1.25 m/s. Estimated MTU contributions are shown for the triceps surae, flexor digitorum and hallucis longus (FDHL), and peroneus longus (*N* = 6).

We computed individual MTU contributions, including contributions from the multiarticular FDHL MTUs (Fig 4). The peak $P_{ank}^{\prime }$ during Push-off was dominated by triceps surae power (89.0 ± 16.7%) while the FDHL and peroneus longus MTUs contributed 6.6 ± 4.1% and 4.4 ± 1.6%, respectively at 1.25 m/s.

**Fig 5:**
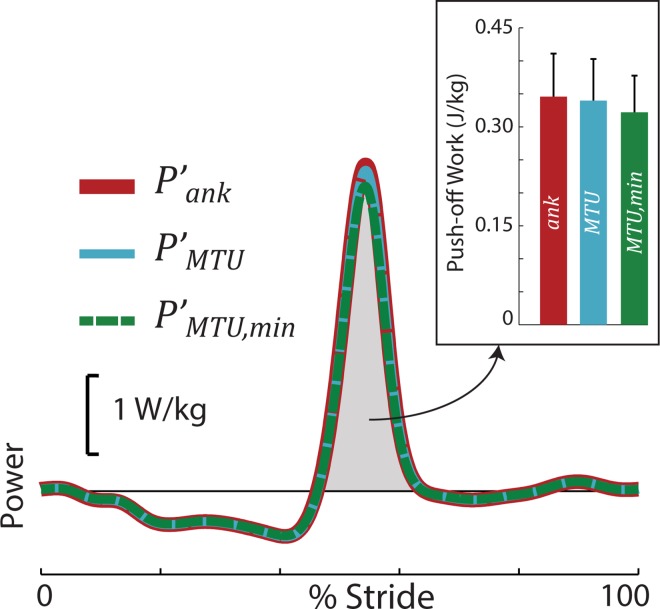
Net ankle power vs. minimum MTU power vs. MTU power during human walking at 1.25 m/s (N = 6). Net ankle power overestimated MTU power and minimum MTU power by about 2% and 7%, respectively, due to multiarticular FDHL dynamics. Inset: Push-off work (area under the power curve within the shaded region) exhibited similar, relatively small differences.

Next, we estimated the minimum plantarflexing MTU power (*P*′_*MTU*,*min*_) and work, by assuming isometric behavior of the FDHL MTUs during Push-off. We found that peak *P*′_*ank*_ overestimated peak *P*′_*MTU*,*min*_ by 7.0% (19.7 ± 10.5 W) at 1.25 m/s. This corresponded with Push-off work differences of 7.4% (1.7 ± 0.8 J, Fig 5). Mean overestimates for peak Push-off power and Push-off work at 0.75 m/s were 4.8% and 5.0%, and at 1.00 m/s were 6.2% and 6.4%, respectively.

We then relaxed the isometric MTU assumption and estimated the net planarflexing MTU power (*P*′_*MTU*_). We found that the FDHL MTUs behaved nearly isometrically, but did lengthen and shorten slightly during Push-off. As such, we found that peak *P*′_*ank*_ overestimated peak *P*′_*MTU*_ by 1.7% (5.1 ± 2.9 W) at 1.25 m/s, which corresponded with 1.8% (0.4 ± 0.3 J) more Push-off work (Fig 5). Mean overestimates for peak Push-off power and Push-off work at 0.75 m/s were 1.2% and 1.3%, and at 1.00 m/s were 1.6% and 1.7%, respectively.

We found that MTU Push-off work estimates were altered by less than 5% when maximum muscle activations were randomly varied by ±50% (*N* = 1). For instance, our sensitivity analysis yielded a range of net plantarflexing MTU Push-off work from 20.8 J to 21.8 J, around the nominal 21.2 J of work at 1.25 m/s. Similar, relatively small effects were observed at other speeds.

## Discussion

We developed an EMG-driven musculoskeletal model to investigate if net ankle joint power overestimates net plantarflexor MTU power during the Push-off phase of human gait due to neglected multiarticular FDHL MTU dynamics. Our EMG-driven model enabled us to estimate individual MTU kinetics during the walking cycle, and account for multiarticular contributions that are not captured by conventional inverse dynamics analysis. We found that ankle power only slightly overestimates net MTU power; nominally by about 2% at 1.25 m/s, but possibly as much as 7% if the FDHL MTUs behave more isometrically than we estimate from motion capture skin markers on the shank and foot. Therefore, over the speeds studied, the FDHL MTU dynamics do not substantially confound the interpretation of net MTU power from inverse dynamics during Push-off. Furthermore, these model predictions were not found to be highly sensitive to uncertainty in maximum EMG activations. This EMG-driven modeling approach may be useful for investigating larger multiarticular MTUs (e.g., hamstrings) and other locomotor activities, in order to improve our multi-scale understanding of movement biomechanics (i.e., linking joint-level biomechanical estimates to our understanding of underlying MTU dynamics).

Various approaches have been taken in the past to characterize muscle- or MTU-specific contributions. One approach assumes that inverse dynamics joint torques are distributed amongst muscles, proportional to their size [34]. However, this approach does not account for muscle-specific activation patterns. A more complex approach involves the generation and analysis of musculoskeletal simulations [14,16–19], which can examine a larger set of muscles, including small or deep muscles that are difficult to measure experimentally. Simulations can be developed using cost function optimizations to solve for muscle activation patterns [16–18], or alternatively can be driven by bio-signals such as EMG [14,19,35]. We employed an EMG-driven musculoskeletal modeling approach of intermediate complexity, which we found to be adequate to estimate MTU-specific contributions about the ankle during the Push-off phase of human gait. Our method utilizes a simple EMG-to-force algorithm, and requires only one scaling and one time delay factor for each subject and speed, which are solved for based on empirical data.

Our EMG-driven model results compared favorably with inverse dynamics estimates, a priori expectations, and with previously published musculoskeletal model results. We observed a strong correlation between our model-estimated power, $P_{ank}^{\prime }$, and inverse dynamics, *P*_*ank*_ (mean R^2^ ≥ 0.97 for all speeds). These correlations were similar to published R^2^ values for a more complex ankle model using Hill-type muscles [36]. We also observed that trends in EMD and the scaling factor were generally consistent with expectations (as summarized in Methods). On average, both EMD and the scaling factor decreased with speed, and EMD remained within a range of values previously reported in literature [37,38]. Additionally, the relative contributions from MTUs were similar to those estimated by musculoskeletal simulations based on Hill-type muscles. To draw this comparison, we used model-based force outputs published in Bogey et al. (2005) to estimate individual MTU power, then compared their relative MTU contributions to our model. This was accomplished by multiplying the published MTU forces by the ankle moment arms and ankle angular velocity used in our study. When comparing our results versus those calculated from Bogey et al. (2005), we found that peak ankle Push-off power was dominated by triceps surae contributions (89.0% vs. 91.6%) and had similar FDHL contributions (6.6% vs. 6.1%).

There are several limitations to the modeling approach we employed, and its generalizability must be further explored and validated. Here we expound upon the limitations and assumptions detailed in the model derivation in the S1 Appendix. We assumed that MTU force production scaled linearly with EMG magnitude at a given speed, since the ankle-foot muscles are known to operate close to isometrically during Push-off at low to moderate speed [39–41]. However, this linear relationship may breakdown for tasks involving a broader combination of eccentric and concentric contractions. It may be necessary to develop new methods for empirically estimating MTU force from bio-signal or imaging modalities. Additionally, we implemented a simplified 2D (sagittal) analysis of forward walking that assumes hinge-like behavior of the ankle, and further work would be needed to extend this approach to 3D analysis. We investigated the implications of neglecting the FDHL MTUs, but did not quantify the effects of other multiarticular muscles that cross the ankle, such as the gastrocnemius or extensor digitorum longus. A final limitation to acknowledge is that our current model estimates net power from each MTU, but does not resolve mechanical power into contributions from muscle fibers vs. tendinous tissues. A grand challenge in the field remains to develop a unified, multi-scale biomechanical understanding, which for instance would enable us to confidently decompose joint-level kinetics into individual MTU kinetics, and then to further decompose these into muscle vs. tendon contributions.

There may be other limitations of inverse dynamics that also cause it to overestimate net MTU power, aside from multiarticular FDHL dynamics. For instance, multisegment foot models have previously shown that the rigid-body foot assumption, common in most inverse dynamics calculations, can lead to an overestimate of peak ankle power by up to 53% (35% on average) due to unmodeled degrees-of-freedom within the foot [42]. Similarly, deformable-body foot estimates indicate that when the ankle and foot are considered together that the peak power is reduced by 25–40% [15,43]. These empirical estimates suggest that the foot may dissipate a significant portion of the Push-off power performed about the ankle, or that peak ankle power is simply overestimated by rigid-body inverse dynamics. Based on the results of this current study, which indicate relatively small contributions from the FDHL MTUs, it does not appear that the previously observed foot absorption can be explained away by unmeasured FDHL dynamics. The functional role of this apparent foot absorption during Push-off in walking remains an open question; and it remains unclear if or how this behavior should be integrated in assistive technologies such as foot prostheses.

The EMG-driven modeling approach presented here may be applied to additional segments and joints within the body to further examine multiarticular MTU contributions during movement. In this study we investigated contributions from relatively small multiarticular MTUs that cross the ankle and toes joints, but further research is needed to understand how larger multiarticular MTUs affect our empirical biomechanical estimates. We focused this study on the FDHL MTUs in order to examine whether their unmodeled dynamics affect our ability to infer net MTU power from joint power during a key phase of the human gait cycle. Muscles such as the gastrocnemius and biceps femoris are larger than the FDHL muscles (in terms of PCSA, [27]), and are expected to contribute more significantly to joint kinetics [44], which may lead to larger discrepancies between joint-level and MTU-level power estimates. This could affect our biomechanical understanding of walking, or other movement tasks. In future investigations, EMG-driven modeling might be extended using complementary measurement modalities. For instance, incorporating B-mode ultrasound could potentially enable us to parse biomechanical contributions from muscles vs. tendons, or ultrasound elastography could provide an alternative way to estimate MTU-specific force [45].

## Conclusions

In summary, we developed and applied an EMG-driven musculoskeletal model to investigate the degree to which net ankle power overestimates net MTU power during the Push-off phase of human walking, due to neglected multiarticular FDHL dynamics. The presented EMG-driven model reproduced ankle joint power with high fidelity, and provided insight on mono- and multi-articular MTU contributions. We found that the behavior of the FDHL MTUs may cause inverse dynamics ankle power to slightly overestimate the net positive power generated by plantarflexion MTUs during Push-off, but only by ~2–7%. This EMG-driven modeling approach could be applied to better account for other multiarticular MTU contributions, such as from the gastrocnemius or biceps femoris, to improve our multi-scale biomechanical understanding of human locomotion.

## Supporting Information

S1 Appendix EMG-Driven Musculoskeletal Model. (PDF)

S1 Fig EMG waveforms at each walking speed. Intersubject mean EMG (solid line) and standard deviation (shaded) for ankle plantarflexor muscles are reported from foot contact to ipsilateral foot contact. Magnitudes are reported as a percentage of maximum muscle activation (N = 6).
(TIF)

S2 Fig Ankle kinematics and kinetics at each walking speed. Depicted are mean sagittal plane ankle kinematics and kinetics (solid line) and standard deviation (shaded) from foot contact to ipsilateral foot contact at 0.75, 1.00 and 1.25 m/s (N = 6). Positive angles and moments represent ankle extension (Ext., plantarflexion) while negative values represent ankle flexion (Flex, dorsiflexion).
(TIF)
