# Supplementary material for: Inferring Muscle-Tendon Unit Power from Ankle Joint Power during the Push-Off Phase of Human Walking: Insights from a Multiarticular EMG-Driven Model
Source: PLoS One. 2016 Oct 20;11(10):e0163169. doi: 10.1371/journal.pone.0163169 (PMC5072599; doi:10.1371/journal.pone.0163169)
Supplement: S1 Appendix — (PDF) [file pone.0163169.s001.pdf]

S1 Appendix: EMG-Driven Musculoskeletal Model

We employed a musculoskeletal model similar to an approach taken by Farris and Sawicki (2012), except that we used an EMG-to-force mapping algorithm rather than assume force was distributed proportionally to muscle size. This modeling approach was used rather than a more complex musculoskeletal simulation (e.g., based on Hill-type muscles), because it required the selection/calibration of fewer unknown parameters. The model was used to gain insights into ankle plantarflexor MTU contributions during the Push-off phase of walking.

We utilized a simple EMG-to-force mapping algorithm to estimate MTU-specific contributions to ankle plantarflexion. For each muscle, m, we used the EMG envelope, muscle size and fiber pennation angle to estimate an unscaled, time-varying force profile, $F'_{m,unsc}$. EMG_m, the normalized EMG signal for each muscle, was defined as a function of time, t, and EMD, τ. Next, the EMG envelope was scaled by the muscle physiological cross sectional area, $PCSA_m$, to account for size differences between muscles ([2], Table a1). Then the EMG envelope was scaled by the cosine of the pennation angle, θ_m, to account for muscle fiber direction, assuming a constant θ_m for each muscle ([2], Table a1). The unscaled force profile ($F'_{m,unsc}$, Eqn. a1) was assumed to be proportional to MTU force [3], but not yet in units of force (e.g., Newtons per kilogram). To obtain a MTU force estimate, F'_m, in units of Newtons per kilogram, we needed an additional subject- and speed-specific scaling factor, C (Eqn. a2).

$$F'_{m,unsc}(t) = PCSA_m \cdot \cos(\theta_m) \cdot EMG_m(t-\tau) \quad \text{(a1)}$$

$$F'_m(t) = PCSA_m \cdot \cos(\theta_m) \cdot EMG_m(t-\tau) \cdot C \quad \text{(a2)}$$

Table a1. Ankle plantarflexion muscle properties and maximum plantarflexion torque.

Muscle	Moment Arm (r_m) (mm) [4]	PCSA (cm^2) [2]	Pennation Angle (θ_m) (°) [2]	Maximum Torque (%)
Soleus	53.1	51.8	28.3	51.9
Medial Gastrocnemius	53.1	21.1	9.9	23.6
Lateral Gastrocnemius	53.1	9.7	12.0	10.8
Flexor Hallucis Longus	24.0	6.9	16.9	3.4
Flexor Digitorum Longus	15.3	4.4	13.6	1.4
Peroneus Brevis	16.7	4.9	11.5	1.7
Peroneus Longus	20.8	10.4	14.1	4.5
Tibialis Posterior	8.9	14.4	13.7	2.7
Total	-	-	-	100.0

The maximum achievable muscle torque ("Maximum Torque" above) was assumed to be proportional to the muscle moment arm [4] multiplied by physiological cross sectional area (PCSA) [2] and the cosine of the pennation angle [2]. Percentages reflect the relative contributions if all muscles were simultaneously maximally contracting.

This simplified EMG-to-force mapping algorithm has several limitations. This analysis ignores the effects of other muscle properties such as the force-velocity and force-length relationships on EMG [5]. Additionally, this analysis ignores the passive structures within the ankle, since

structures such as ligaments act primarily at the limits of the range of motion of the ankle [6]. These angular limits are not reached during normal walking.

We then estimated the unscaled moment profile that each MTU creates about the ankle joint. The unscaled muscle moment , $M'_{m,j,unsc}$ (Eqn. a3, Fig a1), was obtained by multiplying the unscaled force profile, $F'_{m,unsc}$, with the sagittal plane plantarflexion moment arm ($r_{m,j}$, for joint j), estimated from prior cadaveric observation [4]. Moment arms were assumed to be constant, which prior studies suggest is a reasonable first-order approximation given the ankle's range of motion during walking [4,7]. This simplified estimate treats the ankle as a single degree-of-freedom rotational joint, which captures the majority of ankle kinetics during forward walking [8].

$$M'_{m,j,unsc}(t) = r_{m,j} \cdot F'_{m,unsc}(t) \quad \text{(a3)}$$

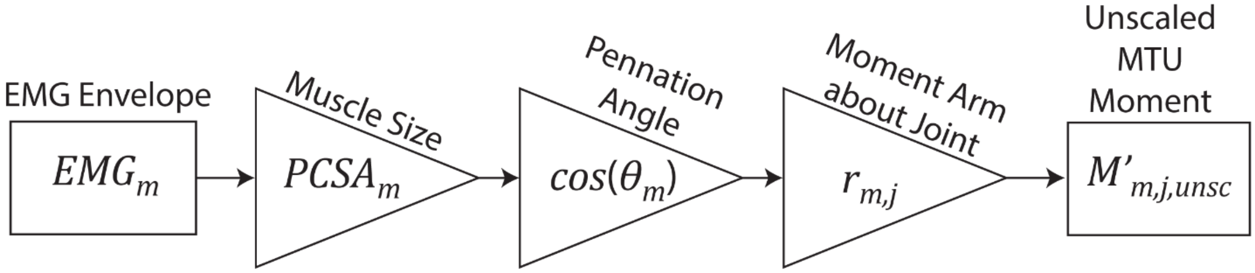

Fig a1. EMG-Driven Model Stage I. For each muscle (or group of muscles) the EMG envelope was multiplied by its physiological cross sectional area ($PCSA_m$), the cosine of the pennation angle (θ_m), and the muscle moment arm about the joint of interest ($r_{m,j}$) – the ankle – to estimate the unscaled MTU moment profile about the ankle ($M'_{m,j,unsc}$). Scaling of this moment profile into units of Nm/kg was performed in Stage II of the model. The boxes indicate a measurement/outcome, the arrows represent a signal/waveform, and the triangles indicate multiplication.

We estimated the net MTU power profile, by summing the MTU-specific moments about the ankle ($\sum M'_{m,j,unsc}$) then multiplying the resultant profile with ankle angular velocity (ω_{ank}), obtained from motion capture (see Fig a2). First, we needed to account for the EMD between muscle activity and mechanical force production [9,10]. To do so, we computed P'_{ank} for varying values of τ (Eqn. a1), then found the EMD that yielded the maximum correlation with sagittal ankle power from inverse dynamics, P_{ank}. We termed this maximally correlated profile as the unscaled ankle plantarflexor power profile ($P'_{ank,unsc}$, Eqn. a4). This power profile is equivalent to summing power estimates form each individual muscle about the ankle ($P'_{m,ank,unsc}$). We assumed the same (constant) EMD for all muscles.

$$P'_{ank,unsc}(t) = \sum M'_{m,ank,unsc}(t) \cdot \omega_{ank}(t) = \sum P'_{m,ank,unsc}(t) \quad \text{(a4)}$$

Next we scaled the magnitude of this model-estimated ankle power, so that it could be directly compared to mass normalized inverse dynamics based estimates (in Watts per kilogram, see Fig

a2). We computed the subject- and speed-specific scaling factor, C, by dividing the peak inverse dynamics sagittal plane ankle power (P_{ank}) by the peak unscaled ankle plantarflexor power profile ($P'_{ank,unsc}$). We assumed that the antagonistic dorsiflexor MTUs perform negligible power during Push-off, which is consistent with the absence of tibialis anterior EMG activity during this phase of gait [11,12]. We assumed that joint friction and foot inertia were also negligible. Additionally, we assumed that our plantarflexor muscle recordings adequately reflected the total plantarflexor contributions, which seemed reasonable given that the set of muscles recorded account for greater than 95% of the plantarflexion moment generating capabilities (see Table a1). Unmeasured plantarflexor muscles were the tibialis posterior, peroneus brevis, and plantaris. The tibialis posterior does have a moderate PCSA [2], but acts at a small (sagittal plane) moment arm [4] compared to the other plantarflexor muscles and exhibits a relatively low EMG activation during gait [13,14], suggesting that its torque contributions are small (Table a1). Similarly, the peroneus brevis is expected to have a relatively small maximum torque contribution to plantarflexion ([12], Table a1). Finally, the plantaris is a small muscle that is only expected to contribute weakly to the ankle plantarflexion moment. It is rarely reported in literature and is in fact a muscle that is absent in about 10% of the population [15]. The resultant model outcome, P'_{ank}, is an EMG-based estimate of ankle power (Eqn. a5); however, as opposed to inverse dynamics, this model-based estimate enables us to parse ankle power into contributions from individual MTUs.

$$P'_{ank}(t) = P'_{ank,unsc}(t) \cdot C = \sum \left(P'_{m,ank,unsc}(t) \cdot C\right) = \sum P'_{m,ank}(t) \qquad \text{(a5)}$$

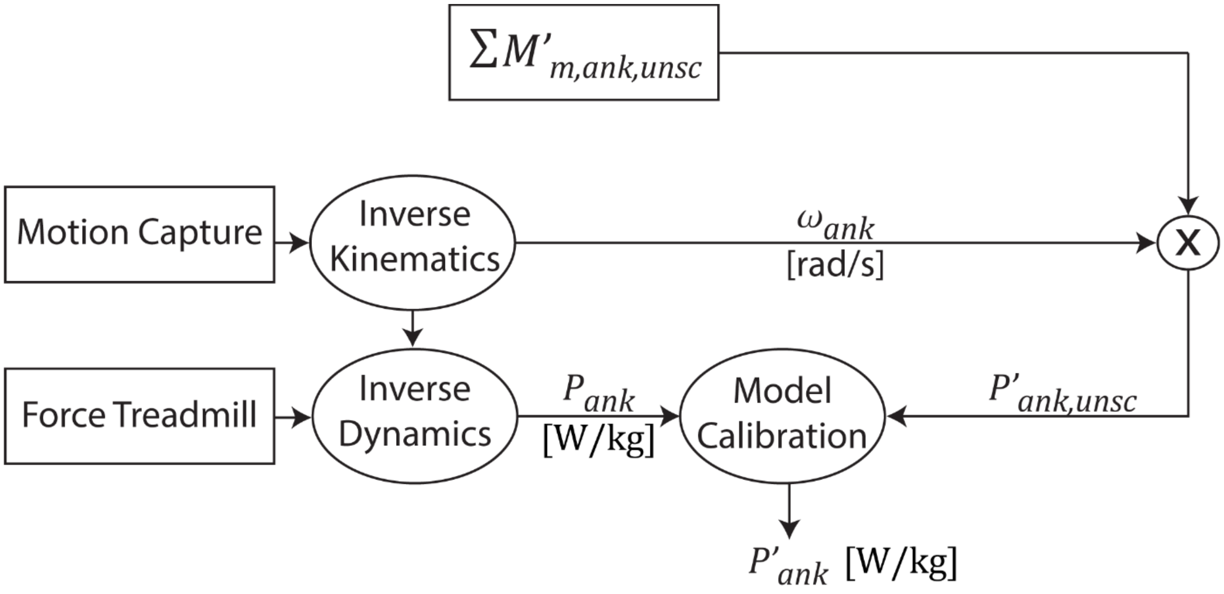

Fig a2. EMG-Driven Model Stage II. The sum of the unscaled muscle moments about the ankle from Stage I was multiplied by sagittal plane ankle angular velocity (ω_{ank}) resulting in an unscaled ankle plantarflexor power profile ($P'_{ank,unsc}$). This profile was then scaled appropriately by matching peak inverse dynamics ankle power (P_{ank}); part of the model calibration. This resulted in an EMG-based estimate of ankle power (P'_{ank}) in units of W/kg. The boxes indicate a measurement/outcome, the arrows represent a signal/waveform, and the ovals represent an algorithm.

References

1. Farris DJ, Sawicki GS. Human medial gastrocnemius force–velocity behavior shifts with locomotion speed and gait. Proc. Natl. Acad. Sci. 2012;109:977–82.

2. Ward SR, Eng CM, Smallwood LH, Lieber RL. Are Current Measurements of Lower Extremity Muscle Architecture Accurate? Clin. Orthop. 2008;467:1074–82.

3. Hof AL. EMG and muscle force: An introduction. Hum. Mov. Sci. 1984;3:119–53.

4. McCullough MBA, Ringleb SI, Arai K, Kitaoka HB, Kaufman KR. Moment Arms of the Ankle Throughout the Range of Motion in Three Planes. Foot Ankle Int. 2011;32:300–6.

5. Buchanan TS, Lloyd DG, Manal K, Besier TF. Neuromusculoskeletal Modeling: Estimation of Muscle Forces and Joint Moments and Movements From Measurements of Neural Command. J. Appl. Biomech. 2004;20:367–95.

6. Winter DA. Biomechanics and Motor Control of Human Movement. John Wiley & Sons; 2009.

7. Zelik KE, Kuo AD. Human walking isn’t all hard work: evidence of soft tissue contributions to energy dissipation and return. J. Exp. Biol. 2010;213:4257–64.

8. Buczek FL, Kepple TM, Siegel KL, Stanhope SJ. Translational and rotational joint power terms in a six degree-of-freedom model of the normal ankle complex. J. Biomech. 1994;27:1447–57.

9. Viitasalo JT, Komi PV. Interrelationships between electromyographic, mechanical, muscle structure and reflex time measurements in man. Acta Physiol. Scand. 1981;111:97–103.

10. Dieën JH van, Thissen CE a. M, Ven AJGM van de, Toussaint HM. The electro-mechanical delay of the erector spinae muscle: influence of rate of force development, fatigue and electrode location. Eur. J. Appl. Physiol. 1991;63:216–22.

11. Winter DA, Yack HJ. EMG profiles during normal human walking: stride-to-stride and inter-subject variability. Electroencephalogr. Clin. Neurophysiol. 1987;67:402–11.

12. Zelik KE, Scaleia VL, Ivanenko YP, Lacquaniti F. Coordination of intrinsic and extrinsic foot muscles during walking. Eur. J. Appl. Physiol. 2014;1–11.

13. Murley GS, Buldt AK, Trump PJ, Wickham JB. Tibialis posterior EMG activity during barefoot walking in people with neutral foot posture. J. Electromyogr. Kinesiol. 2009;19:e69–77.

14. Ringleb SI, Kavros SJ, Kotajarvi BR, Hansen DK, Kitaoka HB, Kaufman KR. Changes in gait associated with acute stage II posterior tibial tendon dysfunction. Gait Posture. 2007;25:555–64.

15. Danforth CH. The Heredity of Unilateral Variations in Man. Genetics. 1924;9:199–211.
