# Supplementary figures and images for: Inferring Muscle-Tendon Unit Power from Ankle Joint Power during the Push-Off Phase of Human Walking: Insights from a Multiarticular EMG-Driven Model
Source: PLoS One. 2016 Oct 20;11(10):e0163169. doi: 10.1371/journal.pone.0163169 (PMC5072599; doi:10.1371/journal.pone.0163169)

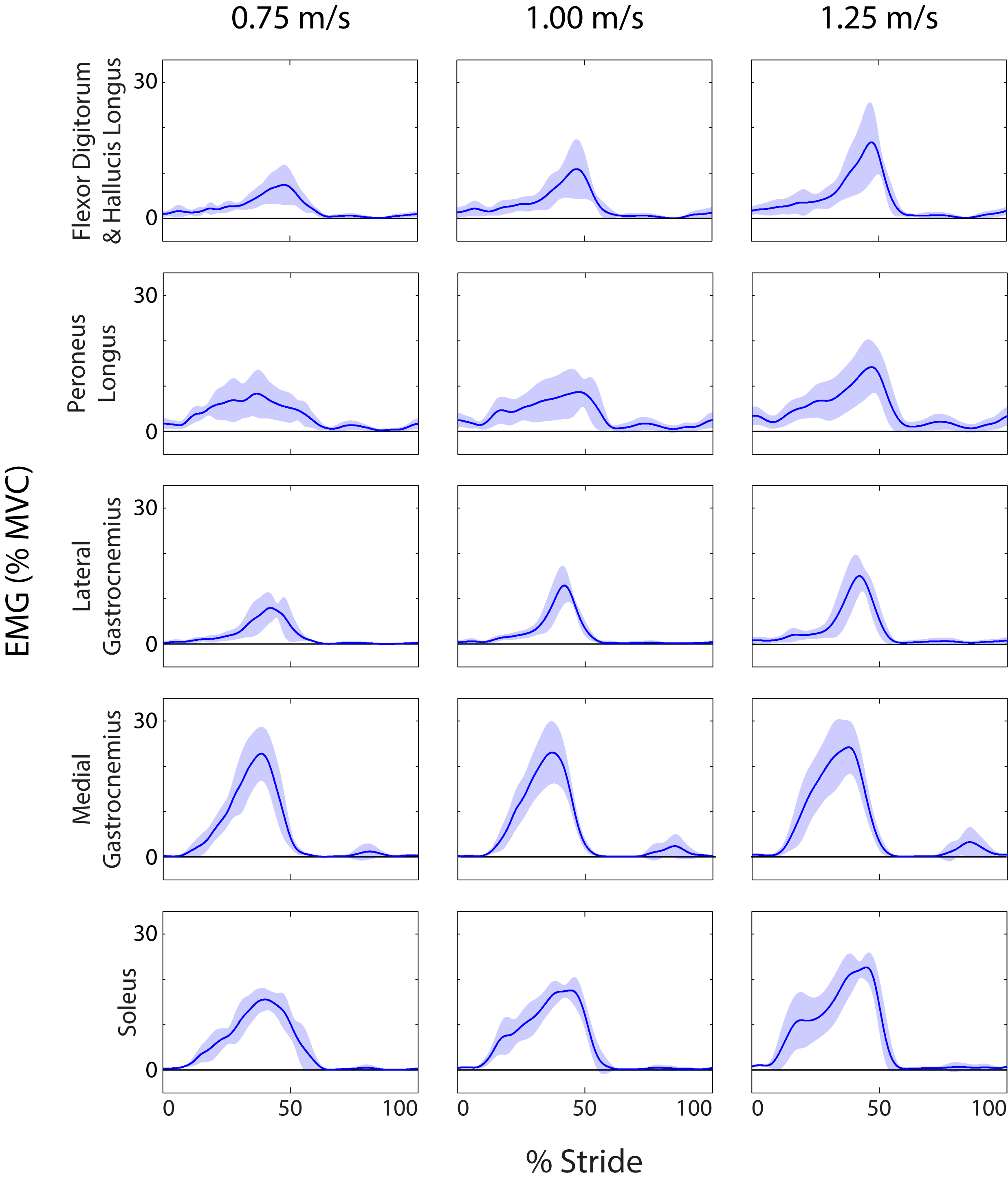

Supplement: S1 Fig — Intersubject mean EMG (solid line) and standard deviation (shaded) for ankle plantarflexor muscles are reported from foot contact to ipsilateral foot contact. Magnitudes are reported as a percentage of maximum muscle activation (N = 6). (TIF) [file pone.0163169.s002.tif]

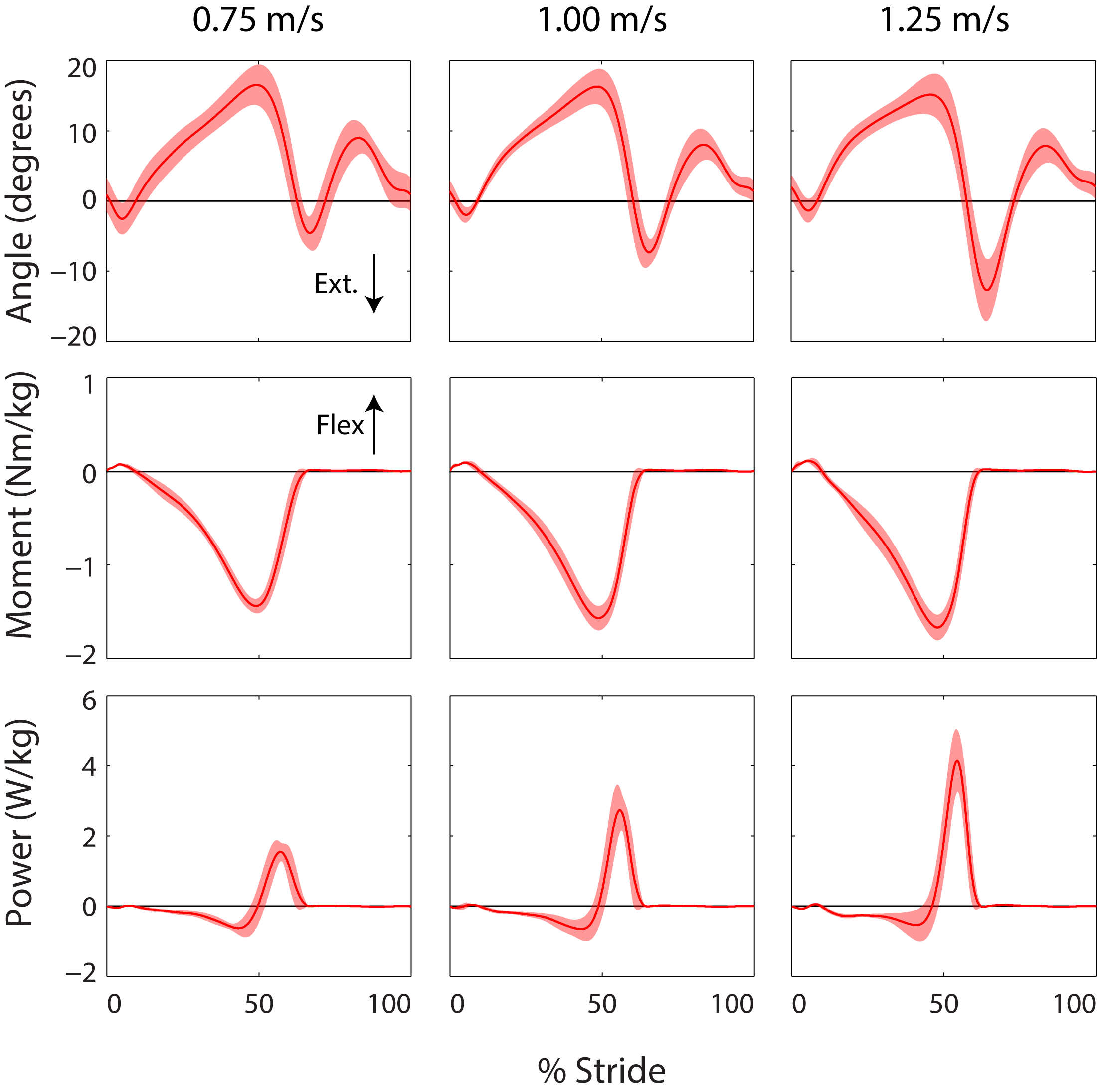

Supplement: S2 Fig — Depicted are mean sagittal plane ankle kinematics and kinetics (solid line) and standard deviation (shaded) from foot contact to ipsilateral foot contact at 0.75, 1.00 and 1.25 m/s (N = 6). Positive angles and moments represent ankle extension (Ext., plantarflexion) while negative values represent ankle flexion (Flex, dorsiflexion). (TIF) [file pone.0163169.s003.tif]
